# Electric-Force Conversion Performance of Si-Based LiNbO_3_ Devices Based on Four Cantilever Beams

**DOI:** 10.3390/mi14111988

**Published:** 2023-10-27

**Authors:** Huiyi Zhang, Xiaojun Qiao, Huifen Wei, Xiaohuang Li, Xiaohui Wu, Nanxin Yu, Hao Lu, Tao Guo, Xiujian Chou, Wenping Geng

**Affiliations:** 1 Science and Technology on Electronic Test and Measurement Laboratory, North University of China, Taiyuan 030051, China; s202106035@st.nuc.edu.cn (H.Z.); xiaojunqiao@nuc.edu.cn (X.Q.); sz202206200@st.nuc.edu.cn (X.L.); sz202106061@st.nuc.edu.cn (X.W.); s202106120@st.nuc.edu.cn (N.Y.); sz202106158@st.nuc.edu.cn (H.L.); guotao6@nuc.edu.cn (T.G.); chouxiujian@nuc.edu.cn (X.C.); 2 Shanxi Provincial Key Laboratory of Advanced Manufacturing Technology, North University of China, Taiyuan 030051, China; b1806002@st.nuc.edu.cn

**Keywords:** lithium niobate, single crystal films, lateral effects, displacement

## Abstract

In micron or nano smart sensing systems, piezoelectric cantilever beams are distributed as major components in microsensors, actuators, and energy harvesters. This paper investigates the performance of four cantilever beam devices with “electric-force” conversion based on the inverse piezoelectric effect of lithium niobate (LiNbO_3_, LN) single-crystal materials. A new compact piezoelectric smart device model is proposed, designed as a single mass block connected by four beams, where devices exhibit smaller lateral errors (0.39–0.41%). The relationship between the displacement characteristics of cantilever beams and driving voltage was researched by applying excitation signals. The results show that the device has the maximum displacement at a first-order intrinsic frequency (*f_osc_* = 11.338 kHz), while the displacement shows a good linear relationship (R^2^ = 0.998) with driving voltage. The square wave signals of the same amplitude have greater “electrical-force” conversion efficiency. The output displacement can reach 12 nm, which is much higher than the output displacement with sinusoidal excitation. In addition, the relative displacement deviation of devices can be maintained within ±1% under multiple cycles of electrical signal loading. The small size, high reliability, and ultra-stability of Si–LN ferroelectric single-crystal cantilever beam devices with lower vibration amplitudes are promising for nanopositioning techniques in microscopy, diagnostics, and high-precision manufacturing applications.

## 1. Introduction

The piezoelectric cantilever beams are critical in designing intelligent systems [1,2,3] as the primary structure of sensors. The cantilever beam structures [4], characterized by wide operating bandwidth, lightweight, powerful driving force, and high electromechanical conversion efficiency, have been used in the fields of micrometer/nanomechanical systems [5,6,7], server hard disk failure detection [8,9] and controller model validation [10,11]. Therefore, the modeling analysis and dynamic motion behavior of piezoelectric cantilever beams have attracted significant works in the literature [12,13].

Lithium niobate (LiNbO_3_, LN) single crystal films have excellent piezoelectric properties and thermal stability (Curie temperature of 1215 °C) [14,15]. In recent years, Qu et al. obtained LN etching patterns with heights of several nm and uniform distribution based on focused ion beam milling [16]. Ying Li et al. applied a proton exchange wet etching technique to remove specific regions of LN films selectively [17]. Jiang et al. completed the etching of the ridge waveguide with a height of 2.5 μm using the proton exchange technique with ion beam-enhanced etching [18]. Huifen Wei et al. implemented a graphical etching technique for 5 μm thick wafer-scale single-crystal films using a dry etching process [19]. With the development of LN etching technology, LN single crystal is expected to become the material of choice for future piezoelectric devices.

Piezoelectric cantilever beams are used in various fields of application due to their interconversion of electrical and mechanical energy. Single cantilever beam structures of piezoelectric devices are used to make energy harvesters [20,21,22]. It has the advantages of simple structure, convenience in fabrication, simple installation, and maintenance [23,24,25,26,27]. Nevertheless, the disadvantages of single cantilever piezoelectric devices are large displacements, residual stresses, and short operating life. Shanky Saxena et al. prepared a novel guided four cantilever beam devices with an output displacement of 95 nm for the input acceleration of 1 g, which is 32.14% smaller than the displacement of single cantilever beams that have been reported [28]. Beyond that, studies on four cantilever beam structures have been reported by Saravanan Shanmugavel et al., who used ferroelectric films of (Pb,La)(Zr,Ti)O_3_ (PLZT) to prepare four cantilever beam structures to study electrical output properties [29]. All of the above-related studies have shown that the four-cantilever beam devices outperform the single-cantilever beam devices in terms of performance and also have excellent fatigue characteristics. However, relatively few reports have been reported on the kinetic characterization of “electric-force” transitions in cantilever beams. Therefore, the analysis of four cantilever beam structural models plays a crucial role in expanding their application areas.

This work investigates the electromechanical response characteristics of cantilever beam structures based on simulated and realized dynamical characteristics. The displacement characteristics of Si-based lithium niobate (LiNbO_3_) ferroelectric single crystal films piezoelectric cantilever beam devices are researched. Firstly, the transverse sensitivity of four-cantilever beam structures is analyzed by simulation. The structure has smaller transverse effects, which can improve the accuracy of devices tested in axial vibration. Secondly, the electrostriction effect of cantilever beam devices with different excitation voltages and the vibration modes of mass blocks are studied by simulation and actual tests, which conclude that the excitation voltages have excellent linearity with the vibration displacements. Finally, the repeatability output performance was tested under different cyclic electrical signal loading. The results show that the four cantilever beam devices possess smaller lateral effects (0.39–0.41%), and the device repeatability error is maintained within ±1%, which contributes to the nanopositioning technology in high-precision equipment having a certain reference value.

## 2. Methods

### 2.1. Mechanism Analysis of Cantilever Beams Vibration

Piezoelectric cantilever beams generally consist of elastic substrates, piezoelectric materials affixed to the elastic substrates, mass blocks, and electrodes, which structure is generally referred to as piezoelectric oscillators. The mass block at the free end is used to reduce the intrinsic frequency of devices, thus ensuring that the devices operate at appropriate frequency bands. In response to the voltage excitation, the piezoelectric cantilever beams will produce bending deformation due to the charge polarization distribution, which results in the vibration of cantilever beams (electrostriction effect). The conversion process of electrical energy to mechanical energy achieved by piezoelectric oscillators is closely related to their mechanical and electrical characteristics, which can be described by the following piezoelectric equation [30,31]:

(1) TpDi=cpqe−dkpdipεiksSqEk

where *T_p_* shows the stress, *S_q_* is the strain, *D_i_* indicates the potential shift, *E_k_* represents the electric field strength, *c* provides the elastic stiffness, *ε* is the dielectric constant, *d* is the piezoelectric constant, *i*, *k* = 1, 2, 3, *p*, *q* = 1–6 indicates the directional sequence number, where the piezoelectric constants of LiNbO_3_ single film material are (*d*_22_ = 20.8, *d*_15_ = 69.2, *d*_31_ = −0.85, *d*_33_ = 6, Unit: pC/N):

(2) $$d=\left[\begin{array}{cc} \begin{array}{ccc} 0 & 0 & 0\\ -d_{22} & d_{22} & 0\\ d_{31} & d_{31} & d_{33} \end{array} & \begin{array}{ccc} 0 & d_{15} & -2d_{22}\\ d_{15} & 0 & 0\\ 0 & 0 & 0 \end{array} \end{array}\right]$$

The system damping [32] during the vibration of the cantilever beam mainly derives from two aspects: one is the air viscoelastic damping [33], during the vibration of piezoelectric oscillators, which can be expressed by viscoelastic damping coefficient *ξ_a_* and the other is strain rate damping [34] during the vibration of piezoelectric oscillators, which can be expressed by strain rate damping coefficient *ξ_s_*. Both of the above damping satisfy the proportional damping criterion [31,35].

The kinetic equations of piezoelectric cantilever beams using the Euler–Bernoulli equation [36] can be expressed as:

(3) $$\frac{\partial^{2}M(x,t)}{\partial x^{2}}+\xi_{s}I_{b}\frac{\partial^{5}y(x,t)}{\partial x^{4}\partial t}+m\frac{\partial^{2}y(x,t)}{\partial t^{2}}+\xi_{a}\frac{\partial y(x,t)}{\partial t}=0$$

where *M*(*x*, *t*) refers to the bending moment of piezoelectric cantilever beams [37], *I_b_* represents the moment of section inertia, and *m* is the mass per unit length. *y*(*x*, *t*) is the absolute vibration displacement of the cantilever beams in the vertical direction, which can be further decomposed into the absolute vibration displacement *y_base_*(*x*, *t*) at the fixed end and the vibration displacement *y_rel_*(*x*, *t*) relative to it:

(4) $$y\left(x,t\right)=y_{base}\left(x,t\right)+y_{rel}\left(x,t\right)$$

Without considering the coupling effect, the bending moment of general cantilever beams is:

(5) $$M(x,t)=EI_{b}\frac{\partial^{2}y_{rel}(x,t)}{\partial t^{2}}$$

where *E* is the modulus of elasticity, *EI_b_* is the bending stiffness of the beam, and there is:

(6) $$EI_{b}=w_{b}\times \frac{Y_{s}\left(h_{b}^{3}-h_{a}^{3}\right)+Y_{p}\left(h_{c}^{3}-h_{b}^{3}\right)}{3}$$

where *Y_s_* is the elastic modulus of substrate material, *Y_p_* shows the elastic modulus of piezoelectric material, *h_a_* represents the distance from the lower surface of the substrate to the neutral axis, *h_b_* demonstrates the distance from the lower surface of the piezoelectric layer to the neutral axis, and *h_c_* indicates the distance from the upper surface of the piezoelectric layer to the neutral axis. However, for piezoelectric cantilever beams, the electromechanical coupling behavior [38] needs to be considered during vibration. In this case, the bending moment is:

(7) $$M\left(x,t\right)=EI_{b}\frac{\partial^{2}y_{rel}\left(x,t\right)}{\partial t^{2}}+\vartheta V_{0}(t)$$

*ϑ* is the coupling term coefficient and has:

(8) $$\vartheta =-\frac{Y_{p}d_{31}w_{b}(h_{c}^{2}-h_{b}^{2})}{2h_{p}}$$

The free vibration equation of the cantilever beam piezoelectric oscillator can be obtained by contacting (3), (4), and (7) as:

(9) $$EI_{b}\frac{\partial^{4}y_{rel}(x,t)}{\partial x^{4}}+\xi_{a}I_{b}\frac{\partial^{2}y_{rel}(x,t)}{\partial x^{4}\partial t}+m\frac{\partial^{2}y_{rel}(x,t)}{\partial t^{2}}+\xi_{a}\frac{\partial y_{rel}(x,t)}{\partial t}+\vartheta V_{0}\left(t\right)=-m\frac{\partial^{2}y_{base}\left(x,t\right)}{\partial t^{2}}-\xi_{a}\frac{\partial y_{base}(x,t)}{\partial t}$$

The absolute vibration displacement at the fixed end can also be expressed as:

(10) $$y_{base}\left(x,t\right)=g\left(x,t\right)+xh(t)$$

The forced vibration equation of the cantilever beam piezoelectric oscillator can be obtained by bringing (10) into (9).

### 2.2. Experimental Design and Simulation

**Figure 1:**
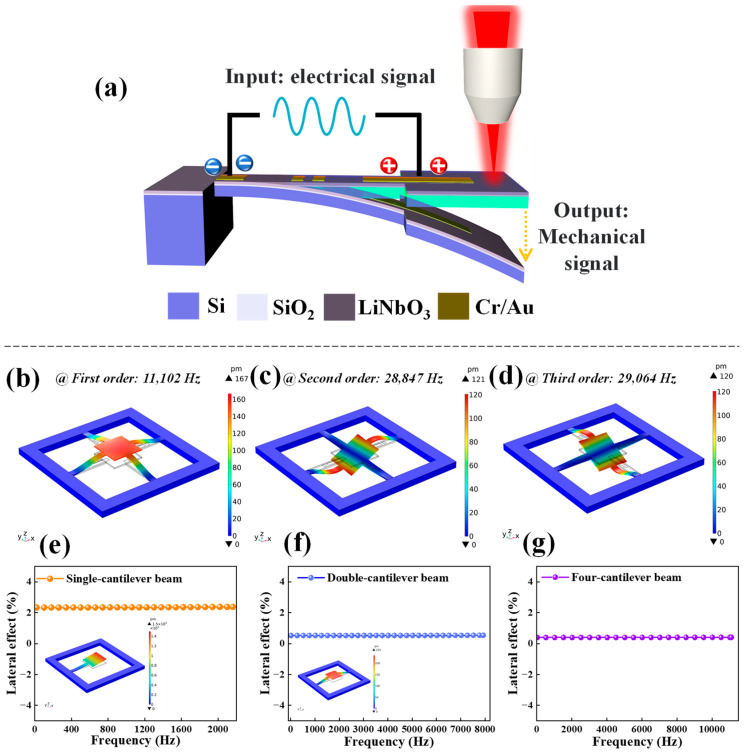
Test principle and simulation calculation. (**a**) Schematic diagram of the conversion from electrical energy to mechanical energy in the quarter-cantilever beam model. (**b**–**d**) Stress distribution cloud at the first three orders of intrinsic frequency when the excitation voltage was set to 10 V. (**e**–**g**) Analysis of lateral effects of single, double, and four cantilever beams structures.

The mechanism of the electrostriction effect on piezoelectric cantilever beam structure is illustrated in Figure 1a; when an electric field is applied at different positions of a piezoelectric crystal, the dielectric will produce mechanical deformation in a certain direction, converting electrical energy into mechanical energy (vibration of cantilever beam), this process can be tested by using Laser Doppler vibrometer (Polytec MSA-400, LDV, Polytek Inc., Waldbronn, Germany).

The dynamics simulation of four cantilever beams structures with COMSOL is shown in Figure 1b–d, representing the first-order bending mode (*f_osc_* = 11,102 Hz) (Figure 1b), second-order twisting mode (*f_osc_* = 28,847 Hz) (Figure 1c) and third-order twisting mode (*f_osc_* = 29,064 Hz) (Figure 1d) stress distribution cloud diagrams, with the parameters of simulation model set as shown in Table 1. Beyond that, the lateral effects of four cantilever structures were analyzed for each order of modalities. The transverse sensitivity of single-cantilever beam, double-cantilever beam, and four-cantilever beam structures are 2.33–2.37%, 0.52–0.54%, and 0.39–0.41% in the first-order intrinsic frequency range (*f_os_*_c_ = 2160.8 Hz, 7923.7 Hz, 11,102 Hz), as shown in Figure 1e–g. A small lateral offset error ensures higher accuracy for smaller displacement outputs in the “electric-force” conversion. The smaller transverse error means that the transverse component of cantilever beams will be minimized when axial strains/stresses are generated, which will improve the testing accuracy of cantilever beam structures in practical applications.

### 2.3. Analysis of Kinetic Characteristics

**Figure 2:**
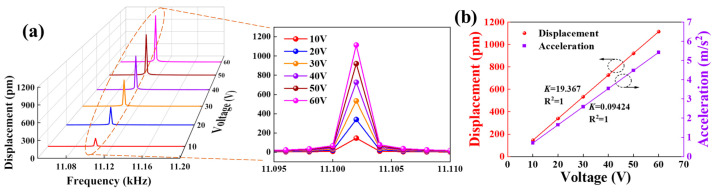
Dynamic simulation analysis of the cantilever beam devices. (**a**) Displacement characteristics with swept signals of different amplitudes. The inset illustrates the localized magnification of the first-order intrinsic frequency in (**a**). (**b**) The dependence of displacement and acceleration at different excitation voltages.

Figure 2 shows four cantilever beam devices’ displacement and acceleration characteristics with different excitation voltages. Figure 2a shows the vibration amplitude at different frequencies when different amplitude excitation voltages are applied, and it can be seen that the intrinsic frequency of devices is 11,102 Hz and increases with the increase of excitation voltage. Figure 2b demonstrates the dependence of displacement and acceleration on excitation voltage, both of which show a positive correlation. The simulation results demonstrate that the ferroelectric single-crystal films have excellent linear output characteristics, which provides a theoretical basis for further experiments.

### 2.4. Devices Preparation Process

**Figure 3:**
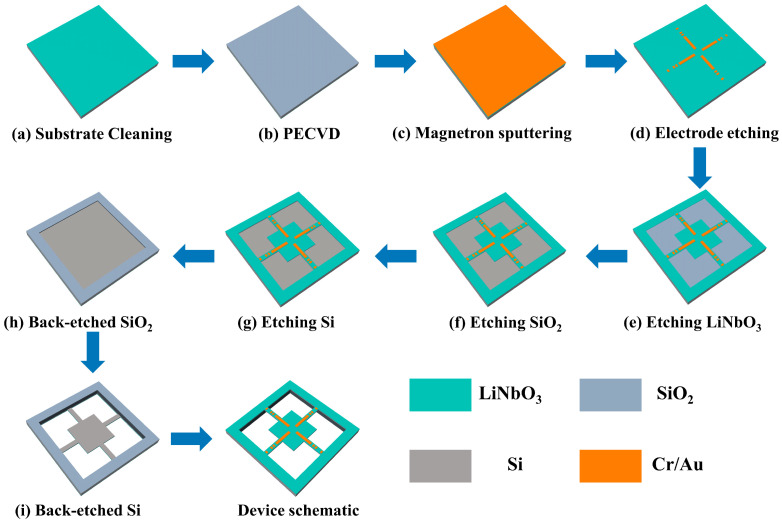
Microstructure manufacturing process.

The fabrication process of four-cantilever beam structure devices is similar to our previous research work [19,39], and the overall dimensions of the device design are 11 mm × 11 mm × 0.5 mm. The preparation process of four cantilever beam devices has been optimized and improved by using a backside SiO_2_ hard mask for backside deep Si etching and the device’s release process to improve the device’s yield. Secondly, the labeling pattern required for the process is prepared with metal electrodes by one-time ion beam etching to shorten the device’s preparation cycle. The microstructure is fabricated by the MEMS process using Si–LN bonding. X-cut lithium niobate (LiNbO_3_, LN) single crystal film samples with thicknesses of 5 μm were used as piezoelectric layers in the devices prepared in experiments. A 2 μm-thick layer of SiO_2_ was grown on the surface of a double-polished wafer, bonded to LN, followed by chemical mechanical polishing (CMP) treatment to obtain a layered LN (5 μm)-SiO_2_ (2 μm)-Si (500 μm) structure, which was purchased from NanoLN Electronic (Jinan, China). The main processes include thermal oxide layer growth, electrode preparation, ion beam etching (IBE), reactive ion etching (RIE), and deep silicon etching processes (DRIE). The fabrication process requires 3 mask plates to complete, with the preparation process shown in Figure 3.

a. Clean the Si-based bonding sheet to ensure clean surfaces (Figure 3a).
b. A 2 μm thick SiO_2_ film is grown on the surface of Si layer for use as a hard pickle film layer when used for Si cavity etching (Figure 3b).
c. The electrodes to be prepared by magnetron sputtering a Cr/Au (20 nm/200 nm) metal layer, followed by photolithography, development and ion beam etching (Figure 3c,d).
d. Ion beam etching technique based on photolithography to complete the etching of 5 μm LiNbO_3_ piezoelectric beams (Figure 3e).
e. Etching of the 2 μm SiO_2_ film is completed on the basis of step d using reactive ion etching technique (Figure 3f).
f. Spraying photoresist on the basis of step e, ensuring adhesive thickness of at least 4 μm, followed by the deep Si etching process to complete Si beam etching with thickness of 100 μm (Figure 3g).
g. Complete 2 μm SiO_2_ film and 400 μm Si substrate etching on the backside of bonded sheets (Figure 3h,i).

## 3. Results and Discussion

**Figure 4:**
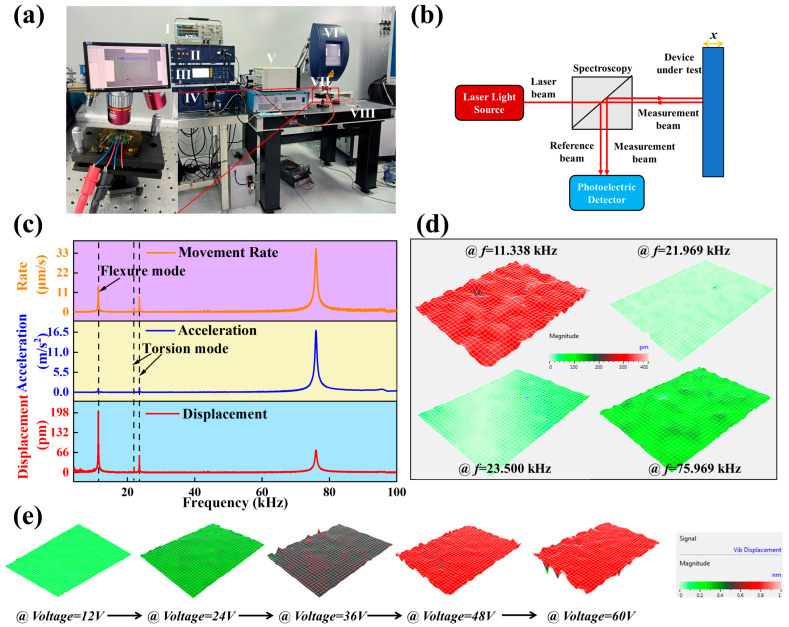
Micro-cantilever beam test system and modal analysis of device structure. (**a**) Laser Doppler test system. Insert shows the laser source performing displacement tests on the center mass. Among them: I: Oscilloscope, II: Junction box, III: Vibrometer Controller, IV: Data management system, V: High voltage Amplifier, VI: Microsystem analyzer, VII: Carrier table, VIII: Vibration Isolator. (**b**) Laser Doppler test principle. (**c**) Variations of vibration amplitude, velocity, and acceleration of the cantilever beam (*f* = 0.1–100 kHz). (**d**) Displacement modal patterns at different eigenfrequencies. (**e**) Vibration displacement modal diagram of the vibration on the mass block surface under different voltage excitation (*f* = 11,338 Hz, *Voltage* = 12 V, 24 V, 36 V, 48 V, 60 V).

The test system of the laser Doppler vibrometer is shown in Figure 4a, which consists of an oscilloscope (Tektronix, Model: DPO 2012 Digital Phoshor Oscilloscope, Tektronix Inc., Beaverton, OR, USA) (for displaying the amplitude and period of output electrical signal), Junction Box, vibration controller (Model: Polytec OFV-5000, Polytek Inc., Waldbronn, Germany), data management system (Model: Polytec DMS, Polytek Inc., Waldbronn, Germany), high voltage amplifier (Model: HA-205, Polytek Inc., Waldbronn, Germany), microsystem analyzer, vibration isolation platform (Model: LSXPT, Polytek Inc., Waldbronn, Germany), and load table. The internal light source optical path of the test system is shown in Figure 4b. Laser Doppler vibrometry is a technique that uses the principle of external differential interference and the Doppler effect to measure the vibration of objects’ surfaces [40]. Based on the principle of external differential interference, the laser with an emitted frequency *f* is split into reference and test beams as it passes through the polarizing beam splitter [41]. Bragg diffraction occurs after the reference beam passes through the acousto-optic modulator, and the frequency of light becomes *f* + *f_AOM_*, *f_AOM_* is the acousto-optic modulation frequency [42]. The test beam is diffusely reflected after passing through the surface of an object, and the reflected frequency becomes *f* + Δ*f*, where Δ*f* is the Doppler shift generated by the object. Subsequently, the two fiber bundles interfere and are received by the photodetector, after which the signals are processed and demodulated.

After the wave from the source reaches a moving object, it is reflected and received by the detector, and the frequency of the received wave changes. The change in frequency (the Doppler shift Δ*f*) can be expressed by the following equation:

(11) $$∆f=\frac{2V}{\lambda }$$

*V* represents the motion velocity of the object, and *λ* is the wavelength of the incident wave since Equation (11) can be derived from Equation (12).

(12) $$V=\frac{\lambda }{2}∆f$$

In principle, LDV can calculate the displacement and acceleration of vibration in addition to the direct measurement of vibration velocity [43]. Assuming that the vibrating object is in simple harmonic motion, its displacement as a function of time *t* can be expressed as [44]:

(13) $$X\left(t\right)=Asin(2\pi ft)$$

where *A* is the amplitude and *f* is the frequency of vibration of the object.

Then, the velocity of the surface of the object is:

(14) $$V\left(t\right)=2\pi f\times Acos(2\pi ft)$$

The acceleration of the vibrating object can be expressed as:

(15) $$a\left(t\right)=-{\left(2\pi f\right)}^{2}\times Asin(2\pi ft)$$

The piezoelectric cantilever beam devices are fixed on microdisplacement stages and loaded with a swept voltage of 15 V for displacement testing in the 0.1–100 kHz frequency band. As shown in Figure 4c, the actual first-order bending mode frequency of the piezoelectric cantilever beam device is 11.338 kHz when the device vibration amplitude is maximum. The vibration velocity and acceleration of cantilever beam devices are maximum over the entire frequency band at frequencies corresponding to the second-order bending of devices. The displacement modal distributions at different intrinsic frequencies for the excitation voltage of 20 V are shown in Figure 4d. Compared to the twisted mode, the bending mode has fewer constraints in the axial vibration and, therefore, is more likely to produce larger values of physical parameters. Table 2 shows the comparison between simulated and actual values of the four cantilever beams. The MEMS manufacturing process cannot be etched precisely, which will cause some deviation.

The electrical energy can be converted into mechanical energy (cantilever beam deformation) by applying voltages to different electrode surfaces according to the electrostrictive effect. Figure 4e shows the modal distribution of displacement on mass block surface with different amplitudes of AC voltage applied; with increasing voltage value, the displacement shows an increasing trend.

**Figure 5:**
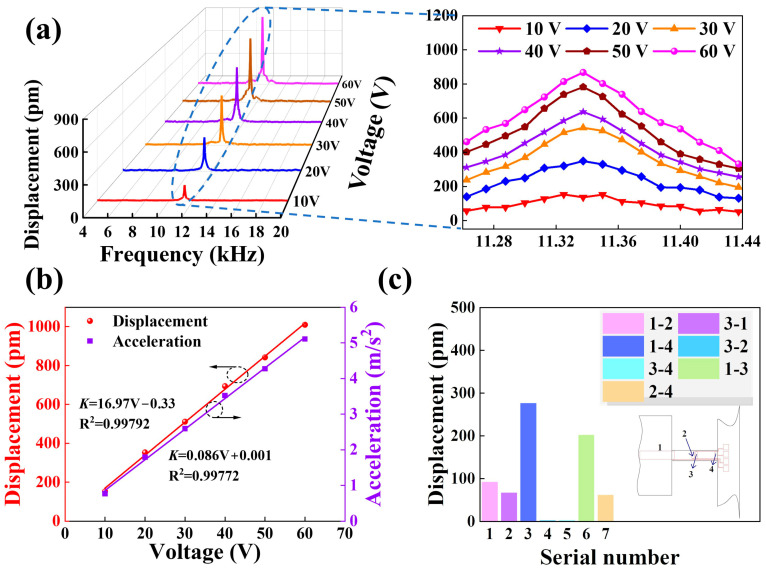
Sweep signal test performance. (**a**) The axial vibration displacement curves of the mass block under 10–60 V input excitation, where the inset is the magnified view of local data. (**b**) Variation of cantilever beam displacement and acceleration under different excitation voltages. (**c**) The displacement response of the cantilever beam when the excitation voltages were applied at different positions. The insert shows the electrodes marked as 1, 2, 3, and 4 in order from mass block position to base position.

Sinusoidal excitation signals of different amplitudes were applied to the cantilever beam structure, with the sweep frequency range set from 0.1 to 20 kHz; the test results are shown in Figure 5a. The maximum displacement value of devices exists at the first-order characteristic frequency, and the vibration amplitude increases with increasing excitation voltage. Figure 5b lists the vibration displacement and acceleration values for the cantilever beam at 11.338 kHz, and the linearity can be achieved above 0.99 with an excellent linear positive growth trend. The actual dynamics tests are in perfect agreement with the simulated results in Figure 2. Beyond that, the mechanical property transitions were studied by applying voltages (*V_in_* = 20 V) at different locations of the beam, as shown in Figure 5c. When the excitation is applied near the free end, it is more likely to produce larger amplitudes.

Figure 2 and Figure 5 provide comparative analyses of the performance of four-cantilever beam devices in terms of “electric-force” conversion from both simulation and experimental perspectives. The displacement response of devices under the action of electrical signals was investigated by applying swept signals with different excitation voltages. In the simulation calculations, the simulated displacement value of the cantilever beam devices is 1.154 nm when the excitation voltage is *V_in_* = 60 V. In the actual test, the actual output displacement value of the cantilever beam device is 0.927 nm when the same type of excitation voltage is applied. Simulations and practical tests show that the displacement response of four cantilever beam devices under the action of electrical signals all increase with the increase of excitation voltages. In addition, we have also analyzed and compared the acceleration characteristics during the vibration of four cantilever beams. Figure 2b and Figure 5b show the dependence of the vibration displacement and acceleration of cantilever beam devices on the excitation voltage at the first-order intrinsic frequency. Under the excitation of AC voltage from 10 V to 60 V, the motion acceleration of devices is between 0 and 7 m/s^2^; when the excitation voltage is 60 V, the simulation calculated value of acceleration is 5.4233 m/s^2^, and the actual test value is 5.1582 m/s^2^, the error value is 0.2651 m/s^2^. At the same time, the vibration displacement and motion acceleration of devices are positively correlated with the excitation voltage with a better linear relationship (R^2^ > 0.99), which implies that LiNbO_3_ single-crystal films have favorable application prospects in the process of “electric-force” conversion. Errors in the simulation and actual test results are unavoidable, which are mainly due to certain differences in the preparation of devices in the various processes. For example, the sleeve etching process does not achieve absolute precision in patterning, and the etching rate and time in the ion beam etching and deep Si etching processes affect the thickness of piezoelectric LiNbO_3_ crystals and Si-based beams, which play key roles in the intrinsic frequency of four cantilever beam devices. In addition, since the tests are based on laser beams to accomplish amplitude measurements for cantilever beam devices, the micro-jitter of test equipment is also critical to the accuracy of tests.

**Figure 6:**
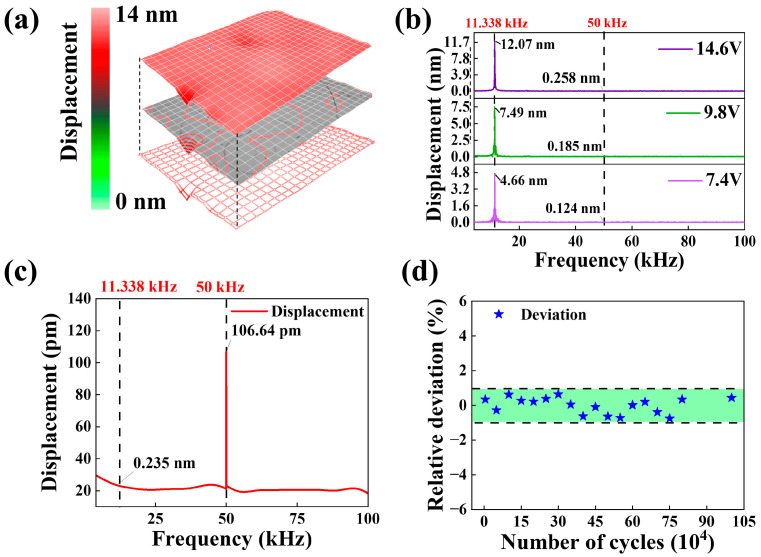
Fixed frequency square wave signal test. (**a**) Displacement modal distribution. (**b**) The displacement values at different voltages under square wave excitation (*f* = 11,338 Hz). (**c**) The value of axial displacement of the mass block is at *f* = 50 kHz (*Voltage* = 7.4 V). (**d**) Repeatability test experiment.

In order to test the mechanical response of cantilever beams at single frequencies, square wave signals of 11.338 kHz were applied to both ends of the cantilever beam. Figure 6a provides the vibration mode diagram of the mass block surface at the *f_os_*_c_ = 11.338 kHz with the square wave signal of amplitude 14.6 V applied. The top layer is the vibration amplitude modal map, the middle layer is the contour map, and the bottom layer is the device scan point map; the results show that the vibration amplitude is about 12.07 nm when the excitation voltage is 14.6 V. The low displacement improves the fatigue resistance of devices and prolongs their operating time, which is promising for nano/pico positioning techniques in microscopic manipulation, medical diagnostics, and high-precision equipment. Figure 6b shows the amplitude response of cantilever beam vibration at 11.338 kHz square wave signal, where the sensors can be separated at this frequency and exhibit large displacement values (compared to the sinusoidal signal); this is because the square wave signal carries more energy than the sinusoidal signal for the same frequency and amplitude excitation, so it can convert higher electrical energy into mechanical energy (inverse piezoelectric effect) during the “electric-force” conversion, which is macroscopically manifested as a significant increase in the amplitude of cantilever beams. Figure 6c demonstrates the displacement distribution at different frequencies under the excitation of the square wave signal at *f* = 50 kHz and *Voltage* = 7.4 V. The results show that the displacement characteristics of devices excited by square wave signals with first-order frequencies are significantly better than the displacement response of square wave signals with non-characteristic frequencies. The reason for this may be that the sinusoidal signal is a single-frequency signal in the “electric-force” conversion process, which will produce a periodic displacement response. The square wave signal as multiple sinusoidal signals superposition (different amplitude, different frequency), while the excitation period (88.2 μs) is less than the response time of devices, which leads to superposition of output displacements. Therefore, the response of devices excited by square wave signals is higher than that of sinusoidal signals. The repeatability of cantilever beams is verified by loading electrical signals (*V_in_* = 20 V, *f* = 11.338 kHz) with different numbers of cycles (see Figure 6d); the relative deviation value of repeatability was kept within ±1%, where lower error values can extend the service life of cantilever beams. Although the device has good fatigue resistance, the cantilever beam structure of sensors is highly susceptible to structural damage and, therefore, still needs to be structurally optimized to ensure that the cantilever beam, in the shorter case, can produce a nanoscale displacement response. The development and miniaturization of MEMS devices demand demanding requirements on the accuracy of high-precision devices. For example, the implementation of nanoscale processes requires photolithography with the capability of line recognition and localization techniques at the nanometer or even picometer level. In patterned microscopy, more precise components are needed to achieve nanometer or even picometer lens offsets. In addition to the rapid development of nano-operated systems/robots, due to their need for very high motion accuracy experiments, nanoscale precision manipulation control, including grasping, lifting, moving, and placing. Transforming the displacement response of four cantilever beams at the nano-/pico-level into operation commands to be input to the execution object is of great application value for realizing high-precision operation.

## 4. Conclusions

In this work, miniature four-cantilever beam piezoelectric structures were prepared based on LiNbO_3_ single-crystal films. By simulating the dynamics of the four-cantilever during vibration, the structure exhibits lower lateral effects (0.39–0.41%). The bonding of the substrate and piezoelectric layer is achieved by the bonding process, which provides high reliability, ultra-stability, miniaturization, and high drive capability. LDV was applied to the piezoelectric beams with different excitation voltages and tested the dynamic characteristics of beams at different positions. The results show that the output displacement and vibratory acceleration show a strong linear relationship with excitation voltage. Displacement properties of LiNbO_3_ single crystal films were analyzed based on swept sine and square wave signals. Square wave signals carry more electrical energy than sinusoidal signals with the same conditions, which can improve the efficiency of “electrical-force” conversion and macroscopically show the greater amplitude of suspension beam displacement. The repeatability deviation of cantilever beams was kept lower (±1%) under cyclic electrical signal loading conditions. The better microdisplacement response points the way for us to carry out the observation of ultraminiaturized structures and the development of broadband microvibration sensors. The devices exhibit wide passband flatness to broaden the application field of piezoelectric cantilever beams, and the nanoscale displacement output characteristics exhibited increase the service life of cantilever beam devices, which has better application value in nanopositioning technology of high-precision instruments.

## Figures and Tables

**Table 1:** The numerical simulation parameters of the device structure.

Symbol	Meaning	Numerical Value
*L_mass_*	The length of the mass block	3600 μm
*W_mass_*	The width of the mass block	460 μm
*L_beam_*	The length of the cantilever beam	2260 μm
*H_beam_*	The height of the cantilever beam	100 μm
*ρ_LN_*	Density of LN	4700 kg/m^3^
*ε_rLN_*	Relative dielectric constants of LN	{43.6, 43.6, 29.16}
*ρ_Si_*	Density of Si	2329 kg/m^3^
*ε_rSi_*	Relative dielectric constants of Si	11.7
*v_Si_*	Poisson’s ratios of Si	0.28
*Y_Si_*	Young’s modulus of Si.	1.7 × 10^11^ Pa
*ρ_Au_*	Density of Au	19,300 kg/m^3^
*v_Au_*	Poisson’s ratios of Au	0.44

**Table 2:** Eigenvalues of the first three orders of modes for four cantilever beam structures.

Mode of Simulation	Measurement Frequency	Description
1st	11.338 kHz	flexure
2nd	21.953 kHz	torsion
3rd	23.547 kHz	torsion
